# S251. QUALITY OF LIFE OF CHRONIC SCHIZOPHRENIA PATIENTS IN THE LONG TERM FOLLOW-UP

**DOI:** 10.1093/schbul/sby018.1038

**Published:** 2018-04-01

**Authors:** Diego Mendes, Gustavo Mustafé, Thalita Fernandes, Thais Martins, Clarissa Dantas

**Affiliations:** University of Campinas

## Abstract

**Background:**

In recent years the goal of the treatment of patients with schizophrenia has shifted from symptom remission to the improvement in health as a whole. In this context, concepts such as quality of life (QoL), social and occupational functioning gained interest. The aim of this study is to investigate changes in QoL of chronic schizophrenia patients in the long-term follow-up and their associations with symptoms and level of community functioning.

**Methods:**

We will contact 85 patients with schizophrenia, considered clinically stable in the previous year, who participated in a study about the deficit syndrome of schizophrenia in 2009/2010. Back then, they were recruited in two sites: an outpatient service of a university general hospital (49 patients) and a community-based service (36 patients). Patients will be assessed with the same instruments adopted in the first study: SAPS, SANS, Calgary Depression Scale and Quality of Life Scale (QLS), plus the Personal and Social Performance Scale (PSP), not used at baseline. We started recruitment by the patients originally treated in the outpatient clinic.

**Results:**

Until now, of the 49 patients, 2 dropped out treatment, 8 had been transferred to other services, 5 refused to participate, 1 had the diagnostic changed to bipolar disorder and 3 had died precociously. Of the deaths, 1 was due to complications secondary to the use of clozapine, 1 due to suicide and 1 patient was murdered by another patient during a psychiatric hospitalization. Up to now, 20 patients completed reassessment, mean age at baseline was 36.9 ± 8.9 years, mean duration of mental illness was 16 ± 10.1 years, and 75% were men. They had in mean, 10.7 ± 3.3 years of education, only 4 had any work activity and 55% had a low socioeconomic position. Assessment interval was 6.9 years ± 0.5. Some demographic aspects slightly worsened: only 15% had an occupation at follow-up, and 60% fell in the lower socioeconomic position. Between assessments, 8 (40%) patients have had periods of noncompliance to medication, 5 had psychiatric hospitalizations, three of them involuntarily. At follow-up 70% of the patients were using clozapine and, despite that, some presented residual positive symptoms (SAPS 6.2 ± 4.8); Calgary mean score was low (2.2 ± 2.2) and, except for the patient who died, there was no new suicide attempts between assessments. Negative symptoms severity was moderate in general (mean SANS 14.8 ± 7.2). Patients as a group had no significant change in QLS scores (61.5 ± 28.2 versus 60.1 ± 28.2 at follow-up). However, 15% of patients had an improvement greater than 20% in their QLS scores, and 30% had a worsening of their scores greater than 20%. Baseline scores on SANS (p=0.005), SAPS (p=0.03) and number of hospitalizations (p=0.03) were negatively correlated with follow-up QLS scores; and years of schooling at baseline was positively correlated (p=0.2). Baseline QLS scores were strongly associated with current QLS scores, as well as with PSP scores (both with P <0.000) at follow-up. Regarding PSP outcomes, 50% of patients were classified in the 70-31 interval (disabilities of various degrees) and 25% were under 30, requiring intensive support.

**Discussion:**

The results presented are partial, obtained with a provisional small sample size. Nevertheless, they show some interesting trends as the possible existence of two patterns of outcome regarding QoL in chronic schizophrenia patients: of significant improvement and one of worsening. If those initial findings are to be confirmed, our next step will be to investigate characteristics associated to improvement or deterioration of QoL in spite of a relative stability of symptoms. That sort of information is of great relevance in the pursuit of recovery for schizophrenia patients.

